# Loss of expression of the recycling receptor, FcRn, promotes tumor cell growth by increasing albumin consumption

**DOI:** 10.18632/oncotarget.13869

**Published:** 2016-12-10

**Authors:** Rafal Swiercz, Min Mo, Priyanka Khare, Zita Schneider, Raimund J Ober, Elizabeth Sally Ward

**Affiliations:** ^1^ Department of Molecular and Cellular Medicine, Texas A&M University Health Science Center, College Station, TX 77843, USA; ^2^ Department of Microbial Pathogenesis and Immunology, Texas A&M University Health Science Center, Bryan, TX 77807, USA; ^3^ Department of Biomedical Engineering, Texas A&M University, College Station, TX 77843, USA

**Keywords:** neonatal Fc receptor (FcRn), albumin, tumor suppressor, recycling receptor, hypoalbuminemia

## Abstract

Tumor cells rely on high concentrations of amino acids to support their growth and proliferation. Although increased macropinocytic uptake and lysosomal degradation of the most abundant serum protein, albumin, in Ras-transformed cells can meet these demands, it is not understood how the majority of tumor cells that express wild type Ras achieve this. In the current study we reveal that the neonatal Fc receptor, FcRn, regulates tumor cell proliferation through the ability to recycle its ligand, albumin. By contrast with normal epithelial cells, we show that human FcRn is present at very low or undetectable levels in the majority of tumor cell lines analyzed. Remarkably, shRNA-mediated ablation of FcRn expression in an FcRn-positive tumor cell line results in a substantial growth increase of tumor xenografts, whereas enforced expression of this receptor by lentiviral transduction has the reverse effect. Moreover, intracellular albumin and glutamate levels are increased by the loss of FcRn-mediated recycling of albumin, combined with hypoalbuminemia in tumor-bearing mice. These studies identify a novel role for FcRn as a suppressor of tumor growth and have implications for the use of this receptor as a prognostic indicator and therapeutic target.

## INTRODUCTION

Cancer cells are characterized by high demands for energy and nutrients to support their rapid proliferation, with glucose and amino acids such as glutamine representing important fuels for tumor cell growth [1]. In tumors that are Ras-transformed, the requirement for amino acids can be met by significantly increased macropinocytic uptake of proteins, with the most abundant serum protein, albumin, playing an important role as a nutrient source [2, 3]. However, the majority of tumors are not Ras-transformed [4], leading to the question as to how these tumors acquire sufficient amino acids to support their high proliferative rates.

Following internalization into tumor cells, albumin undergoes proteolytic degradation in lysosomes to provide the requisite amino acids [2]. The importance of albumin as a nutrient source for tumors, combined with the knowledge that cancer patients are frequently hypoalbuminemic [5], suggests that in the absence of oncogenic Ras mutations such tumors may have alterations in other pathways to enable increased albumin delivery to degradative compartments. The recycling neonatal Fc receptor, FcRn, is expressed in multiple cell types, including normal epithelial cells [6, 7], and represents a candidate receptor for the regulation of the levels of proteins and their constituent amino acids within cells. This receptor interacts with both albumin and antibodies of the immunoglobulin G (IgG) class in a highly pH-dependent way, with binding at acidic pH (~pH 6.0) that becomes negligible at near neutral pH [8, 9]. This pH dependence allows FcRn to salvage its ligands from lysosomal degradation within cells by mediating their transport from acidic sorting (early) endosomes into the recycling or transcytotic pathway followed by exocytic release [9–12]. Consequently, FcRn limits the amount of albumin that is degraded within cells. This motivates the underlying hypothesis for the current study, namely that the loss of FcRn expression in tumor cells increases the availability of albumin-derived amino acids, thereby promoting tumor growth.

To investigate a role for FcRn in regulating the delivery of nutrients to tumor cells, we have analyzed the expression and functional effects of this receptor on the behavior of breast and prostate cancer cells. Consistent with recent analyses of the expression of this receptor in lung cancer [13], the majority of tumor cell lines have very low to undetectable levels of FcRn. Importantly, we demonstrate that the growth of tumors in xenograft models is significantly higher for cells with lower, or knocked down, expression of FcRn. In addition, the specificity of these effects has been shown using a mutated variant of FcRn [14] that does not interact with albumin. Mice bearing tumors that do not recycle albumin due to FcRn deficiency or expression of mutated FcRn are hypoalbuminemic. Collectively, our observations reveal that FcRn can act as a metabolic regulator to suppress tumor cell growth and proliferation.

## RESULTS

### The majority of breast and prostate tumor cell lines express functional FcRn at undetectable or very low levels

**Figure 1:**
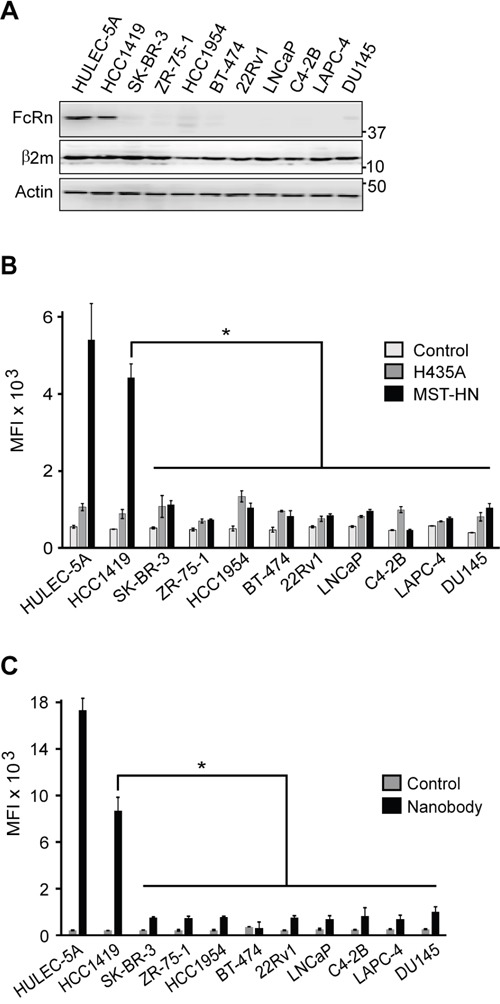
The majority of breast and prostate tumor cell lines express very low or undetectable levels of FcRn **A**. Immunoblotting of cell lysates using antibodies specific for FcRn α-chain, β_2_-microglobulin (β_2_m) and β-actin. Cropped images are shown with molecular weights (kDa) on the right. **B**. Cells were pulsed with 10 μg/ml Alexa 647-labeled MST-HN or Alexa 647-labeled H435A for 40 minutes at 37°C in medium (pH 6.0). Alexa 647 levels (mean fluorescence intensity, MFI) were determined using flow cytometry and mean values for triplicate samples are shown. Control indicates autofluorescence levels of cells. **C**. Cell lines were pulsed with 1 μg/ml Alexa 647-labeled FcRn-specific (bivalent) nanobody for 40 minutes at 37°C in medium (pH 6.0) and analyzed using flow cytometry as in panel (B). Control indicates autofluorescence levels of cells. For panels (B) and (C), error bars represent S.D. Significant differences between MST-HN or nanobody levels are indicated by * (one-way ANOVA, p < 0.05). Data are representative of at least two independent experiments.

We first investigated the levels of FcRn in a total of ten breast and prostate tumor cell lines by immunoblotting with an FcRn α-chain-specific antibody (Figure 1A). All of these tumor cell lines express wild type Ras [15]. Nine of the cell lines have either undetectable, or very low, levels of FcRn (Figure 1A). Notably, the breast cancer cell line HCC1419 exhibited substantially higher expression of FcRn, with a similar level to that of the microvasculature-derived endothelial cell line, HULEC-5A. In earlier studies, this endothelial cell line has been shown to efficiently recycle the FcRn ligand, IgG [16]. We also analyzed the levels of expression of β_2_-microglobulin (β_2_m) which is an obligate partner for egress from the endoplasmic reticulum, stabilization and functional activity of FcRn α-chain during biosynthesis [17]. By contrast with FcRn, the levels of β_2_m were either not affected or reduced slightly (Figure 1A and Supplementary Figure S1), indicating that the substantial decreases in FcRn expression in the tumor cell lines are not due to β_2_m loss.

To investigate the levels of functional FcRn in the tumor cell lines, the accumulation of a fluorescently labeled, engineered antibody (MST-HN; human IgG1-derived) that binds to FcRn with increased affinity in the pH range 6.0-7.4 within cells [18] was investigated (Figure 1B). The levels of MST-HN were compared with those of a mutated antibody (H435A) that does not interact with human FcRn [19], and the results are congruent with the immunoblotting analyses. In addition, the expression data for the different cell lines were confirmed using a nanobody (camelid VHH domain) that binds with high affinity to human FcRn (Figure 1C). For 9 of the 10 cell lines, the levels of FcRn within the cells are also consistent with mRNA levels reported in the Cancer Cell Line Encyclopedia database ([15]; LAPC-4 was not included).

### Albumin accumulation in tumor cells is dependent on FcRn levels

**Figure 2:**
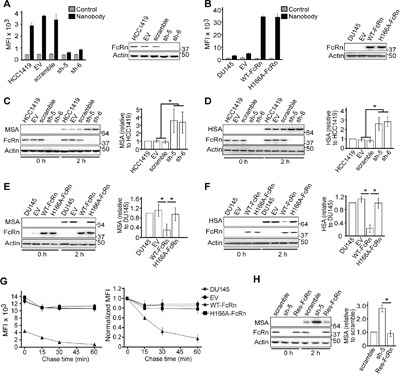
The level of FcRn expression regulates the intracellular levels of albumin in tumor cells **A**. HCC1419 cells transduced with empty vector (EV), scrambled shRNA (scramble) or shRNAs targeting FcRn (sh-5, sh-6) were pulsed with 1 μg/ml Alexa 647-labeled FcRn-specific nanobody for 40 minutes at 37°C in medium (pH 6.0). Alexa 647 levels (MFI) were determined using flow cytometry and mean values for triplicate samples are shown. Control indicates autofluorescence levels of cells. The right hand panel shows immunoblotting of cell lysates using antibodies specific for FcRn α-chain and β-actin. **B**. DU145 cell lines transduced with empty vector (EV) and FcRn expression constructs (WT-FcRn or H166A-FcRn) were analyzed as in panel (A). **C, D**. HCC1419 cell lines (same as in panel A) were pulsed with 1.5 μM (100 μg/ml) MSA (C) or 1.5 μM (100 μg/ml) HSA (D) for 0 h or 2 hrs at 37°C at pH 7.4. Cell lysates were used in immunoblotting with antibodies specific for albumin (MSA/HSA), FcRn α-chain and β-actin. Albumin levels were quantitated and normalized relative to HCC1419 cells. **E, F**. DU145 cell lines (same as in panel B) were treated with MSA (E) or HSA (F) as in panels (C) and (D), and albumin levels quantitated and normalized relative to DU145 cells. **G**. DU145 cell lines were pulsed with 1.5 μM Alexa 647-labeled MSA for 2 hrs. Cells were chased for the indicated times. Alexa 647 levels (MFI) were determined using flow cytometry and mean values for triplicate samples are shown (left panel). The right hand panel shows the MFI values normalized to the no chase (0 time point) MFI value for each cell line. **H**. HCC1419 cell lines transduced with scrambled shRNA (scramble), sh-5 or sh-5 plus shRNA resistant FcRn (Res-FcRn) were treated and albumin levels relative to ‘scramble’ analyzed as in panel (C). For panels (C-F) and (H), mean normalized signal levels derived from three independent immunoblotting experiments are shown. For the immunoblots, cropped images are shown with molecular weights (kDa) on the right. For panels (A-H), error bars represent S.D. Significant differences are indicated by * (one-way ANOVA, *p* < 0.05). For panels (A, B) and (G), data are representative of at least two independent experiments.

The substantially higher levels of FcRn in HCC1419 cells compared with the other breast and prostate cancer cell lines analyzed (Figure 1) provided us with a tool to investigate the effect of reduced FcRn expression on tumor cell line behavior. By comparison with HCC1419 cells lentivirally transduced with vector alone (‘empty vector’) or scrambled shRNA, transduction with two FcRn-specific shRNAs (sh-5, sh-6) substantially reduced FcRn expression (Figure 2A). Reciprocally, to assess the effect of increased FcRn expression levels, the prostate cancer cell line, DU145, was lentivirally transduced with two different human FcRn-expressing constructs: wild type FcRn (WT-FcRn) and a mutated FcRn variant (H166A-FcRn) that has lost binding to albumin with retention of affinity for IgG [14]. DU145 cells transduced with WT-FcRn or H166A-FcRn were cloned and lines with similar FcRn expression levels were used throughout these studies (#2 for each; Figure 2B and Supplementary Figure S2A). Importantly, these clonal lines showed similar behavior to that of their parent polyclonal lines (data not shown).

The accumulation of mouse or human serum albumin (MSA or HSA, respectively) over a two hour incubation period in the different cell lines was determined by immunoblotting (Figure 2C-2F). Knockdown of FcRn in HCC1419 cells resulted in ~3-fold increases in intracellular albumin levels (Figure 2C, 2D), whereas elevated expression of FcRn in DU145 cells reduced albumin accumulation by 3-4 fold (Figure 2E, 2F). Further, the levels of albumin in DU145 cells expressing H166A-FcRn were increased relative to those in WT-FcRn/DU145 cells, and were indistinguishable from those in DU145 cells transduced with empty vector (Figure 2E, 2F). Similar results were obtained following 1 or 4 hours incubation of cells with albumin, except that lower or higher levels of albumin accumulation, respectively, were detected in the FcRn-expressing cells (Supplementary Figure S2B, S2C). Consistent with the immunoblotting data, WT-FcRn/DU145 cells showed higher activity in albumin recycling assays compared with DU145 cells or DU145 cells transduced with H166A-FcRn or empty vector (Figure 2G). Importantly, the phenotype of sh-5 transduced HCC1419 cells was rescued by transduction with an expression construct containing an FcRn gene with silent mutations (Res-FcRn) to confer resistance to shRNA targeting (Figure 2H).

**Figure 3:**
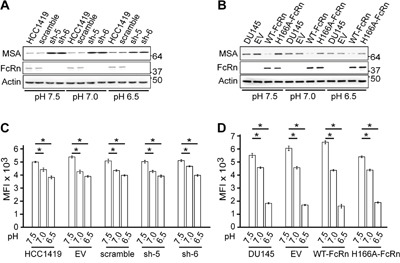
Decreased pH affects fluid phase uptake by the cell lines **A, B**. HCC1419 and DU145 cell lines were treated with MSA as in Figure 2C, except that the incubation medium was adjusted to the indicated pH. Immunoblotting of cell lysates using antibodies specific for FcRn α-chain and β-actin was performed. Cropped images are shown with molecular weights (kDa) on the right. **C, D**. HCC1419 and DU145 cell lines were pulsed with 1.5 μM Alexa 647-labeled H435A for 2 hrs in medium adjusted to the indicated pH. Alexa 647 levels (MFI) were determined using flow cytometry and mean values for triplicate samples are shown. Error bars represent S.D. Significant differences are indicated by * (one-way ANOVA, *p* < 0.05). For panels (A-D), data are representative of two independent experiments.

The pH dependence of the albumin-FcRn interaction (binding at pH 6.0 with no detectable interaction at near neutral pH [8]), combined with the observation that the tumor microenvironment can be acidic (pH 6.5-6.9 [20]), prompted us to also investigate the effect of pH (pH 6.5-7.5) on albumin accumulation in HCC1419 and DU145 cells (Figure 3A, 3B). These data indicate similar inhibitory effects of FcRn expression on intracellular albumin levels in the pH range 6.5-7.5. However, we observed lower levels of albumin accumulation in cells as the pH was lowered, prompting an investigation of pinocytic activity of the cells under different pH conditions. Analyses of the accumulation of the mutated IgG variant, H435A [19], that enters cells by fluid phase uptake, indicated lower levels of cell-associated protein as the pH was decreased (Figure 3C, 3D). Interestingly, this effect was more marked for DU145 compared with HCC1419 cells. These observations indicate that acidic pH reduces the pinocytic activity of the tumor cells.

**Figure 4:**
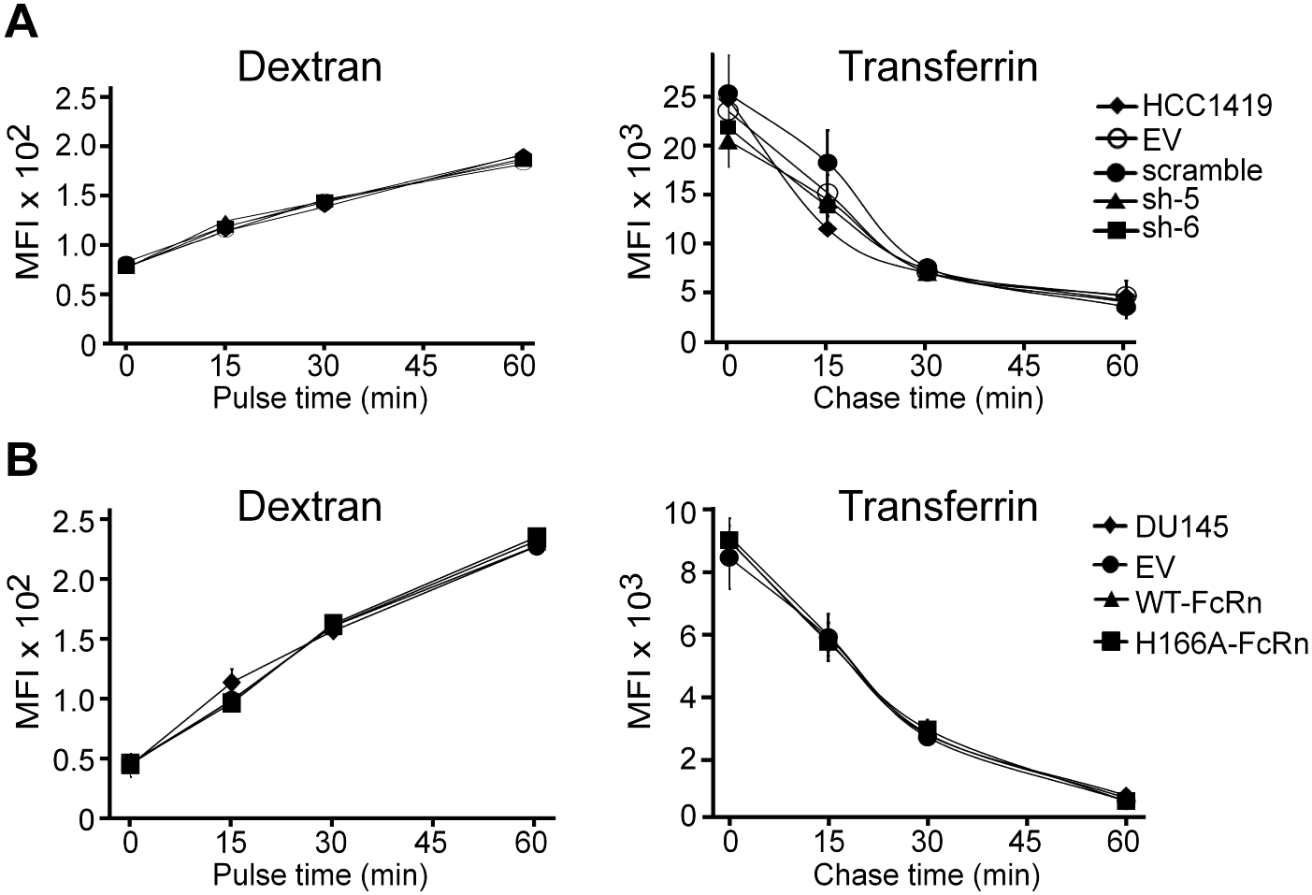
Pinocytic uptake and recycling rates of tumor cells are not affected by lentiviral transduction or the level of FcRn expression **A**. HCC1419 cell lines transduced with empty vector (EV), scrambled shRNA (scramble) or shRNAs (sh-5, sh-6) were pulsed for 0-60 minutes with 1.5 μM tetramethylrhodamine-labeled 70 kDa dextran, or pulsed for 5 minutes with 20 μg/ml Alexa 647-labeled transferrin followed by chase periods of 0-60 minutes. **B**. DU145 cell lines transduced with empty vector (EV) or FcRn expression constructs (WT-FcRn or H166A-FcRn) were treated with Alexa 647-labeled dextran or Alexa 647-labeled transferrin as in panel (A). Flow cytometry was used to determine cell-associated fluorescence. The MFI values represent means derived from triplicate samples. Error bars represent S.D. Data are representative of two independent experiments.

It was important to ensure that the differences in albumin accumulation were not due to alterations in fluid phase pinocytic and/or recycling activity of the transduced cell lines. Flow cytometry was therefore used to quantitate the fluid phase uptake of Alexa 647-labeled dextran (70 kDa) and recycling of Alexa 647-labeled transferrin (Figure 4). These analyses demonstrated that increased FcRn expression or knockdown in DU145 and HCC1419 cells, respectively, does not have non-specific effects on pinocytic or recycling activity.

### Tumor growth in mouse xenograft models is dependent on FcRn levels

**Figure 5:**
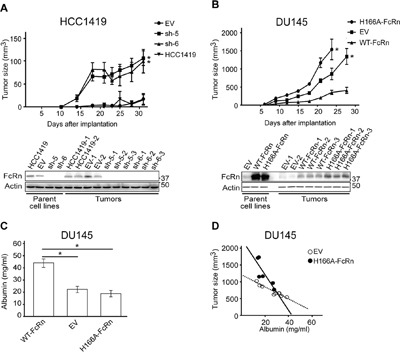
The expression level of FcRn controls the growth of tumor xenografts **A**. Mice (n = 6-8 mice/group) were implanted with HCC1419 cells transduced with shRNAs targeting FcRn (sh-5, sh-6) or empty vector (EV) and tumor size monitored (upper panel). Statistically significant differences between the EV group and knockdown (sh-5, sh-6) groups from days 18-31 are indicated by * (two-way ANOVA with Tukey post-hoc comparison; *p* < 0.05). Immunoblotting analysis of lysates of tumors (HCC1419 cell lines) extracted at the end of the experiment using antibodies specific for human FcRn α-chain and β-actin is shown in the lower panel. Cropped images are shown with molecular weights (kDa) on the right. Data are representative of two independent assays. **B**. Mice (n = 9-10 mice/group) were implanted with DU145 cells transduced with FcRn expression constructs (WT-FcRn or H166A-FcRn) or empty vector (EV) and tumor size monitored (upper panel). Statistically significant differences from days 21-28 (EV vs. WT-FcRn) and days 21-24 (H166A-FcRn vs. WT-FcRn) are indicated by * (two-way ANOVA with Tukey post-hoc comparison; *p* < 0.05). Immunoblotting analysis of lysates of tumors (DU145 cell lines) was carried out and displayed as described in panel (A). **C**. Serum albumin levels at day 21 for mice within different groups for experiment shown in panel (B). **D**. Plot of tumor size vs. serum albumin levels (day 21) for mice implanted with DU145 cells transduced with EV or H166A-FcRn. Statistically significant differences between groups in panel (C) are indicated by * (one-way ANOVA, *p* < 0.05). Data are representative of at least two independent experiments.

The effects of FcRn knockdown or increased expression on albumin accumulation within the tumor cell lines suggested that the levels of FcRn would affect growth as tumor xenografts. BALB/c scid mice were therefore implanted with HCC1419 or DU145 cell lines. Remarkably, FcRn knockdown in HCC1419 cells (sh-5, sh-6) resulted in tumor growth, whereas implantation with wild type HCC1419 cells or HCC1419 cells transduced with empty vector resulted in either undetectable, or very small tumors (Figure 5A). Reciprocally, DU145 cells transduced with WT-FcRn grew significantly more slowly than the corresponding controls, including DU145 cells expressing H166A-FcRn (Figure 5B). Immunoblotting analyses of tumors isolated at the end of the experiments indicated the expected levels of FcRn α-chain for both HCC1419 and DU145 cell lines (Figure 5A, 5B). FcRn expression was lower in WT-FcRn/DU145 cells and H166A-FcRn/DU145 cells compared with the parent cell lines, most likely due to the presence of other cells of mouse origin in the tumors. The immunoblotting results were confirmed for the HCC1419 cell lines using immunohistochemistry (Supplementary Figure S3). In addition, for both HCC1419 and DU145 cells, the levels of accumulated mouse albumin in the tumors were decreased by expression of wild type FcRn (Supplementary Figure S4).

### Serum albumin levels are decreased in mice bearing FcRn-deficient tumors

We next investigated whether increased tumor growth rates led to lower serum albumin levels in mice. Serum alanine aminotransferase (ALT) levels that were greater than 2.5-fold above normal indicated liver dysfunction with possible effects on albumin biosynthesis, and the corresponding mice (4 in H166A-FcRn group; 1 in empty vector group) were therefore excluded from these analyses. Significantly higher levels of serum albumin were observed in mice implanted with WT-FcRn/DU145 tumors relative to those bearing tumors derived from empty vector/DU145 or H166A-FcRn/DU145 cells (Figure 5C). Further, the inverse correlation between tumor size and albumin levels (correlation coefficient of -0.96 and -0.84 for EV and H166A-FcRn, respectively; the slopes of the correlation plots for the two groups are not significantly different, *p* = 0.142) indicates that tumor consumption contributes to the reduced serum albumin concentrations (Figure 5D).

### Intracellular glutamate levels and proliferation of the tumor cells *in vitro* are consistent with xenograft behavior

**Figure 6:**
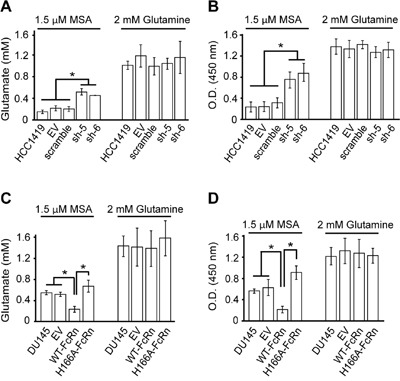
The expression level of FcRn regulates intracellular glutamate levels and cell proliferation **A**. HCC1419 cells transduced with empty vector (EV), scrambled shRNA (scramble) or shRNAs targeting FcRn (sh-5, sh-6) were cultured in base medium containing 100 μM glucose and 1.5 μM MSA or 2 mM glutamine for 24 hrs. Cells were lysed and glutamate levels determined. **B**. HCC1419 cell lines were cultured in the same medium as for panel A for 16 hours. BrdU levels in cells were determined following 4 hrs incubation with BrdU. **C, D**. DU145 cells transduced with empty vector (EV) or FcRn expression constructs (WT-FcRn or H166A-FcRn) were treated as in panels A and B to assess intracellular glutamate levels and proliferation, respectively. For panels A-D, mean values for triplicate samples are shown. Error bars represent S.D. Significant differences are indicated by * (one-way ANOVA, *p* < 0.05). Data are representative of at least two independent experiments.

To further investigate the behavior of the tumor cell lines, the effect of FcRn expression on glutamate accumulation and proliferation were analyzed using *in vitro* assays in the presence of albumin as sole amino acid source. Under these conditions, glutamate can be used as an indicator of albumin uptake and lysosomal degradation [2]. Knockdown of FcRn in HCC1419 cells resulted in increased intracellular glutamate and proliferation (Figure 6A, 6B). By contrast, DU145 cells expressing WT-FcRn exhibited lower levels of intracellular glutamate and proliferation, and this phenotype was reversed by expression of H166A-FcRn (Figure 6C, 6D). Importantly, all HCC1419 and DU145 cell lines had similar levels of glutamate and proliferated at the same rate when albumin was replaced by 2 mM glutamine (Figure 6A-6D). In addition, the effect of FcRn knockdown in HCC1419 cells was reversed by lentiviral transduction with the shRNA-resistant plasmid, Res-FcRn (Supplementary Figure S5). Thus, and consistent with the xenograft experiments, FcRn expression in tumor cells reduces glutamate accumulation and proliferation *in vitro* when albumin is provided as an amino acid source.

## DISCUSSION

The high energetic and biosynthetic requirements of tumor cells drive the metabolic reprogramming that is a hallmark of cancer. Paradoxically, these demands are stressed further by the limited availability of nutrients in the tumor microenvironment [3, 21]. Consequently, alterations of pathways affecting metabolism in tumor cells to compensate for the unfavorable microenvironment are common [22]. For example, the enhanced macropinocytic activity of Ras-mutated cells increases uptake of extracellular proteins such as albumin, allowing these cells to survive under conditions of low amino acid (glutamine) availability [2]. However, whether tumor cells that do not express oncogenic Ras proteins, constituting about 70% of tumors [4], have compensatory processes to enhance protein (albumin) acquisition is unexplored. Here we have identified a novel mode of metabolic regulation operating at the level of the endosomal recycling pathway in such tumor cells. Specifically, reduced expression of the recycling receptor, FcRn, results in increased tumor cell growth both *in vitro* and in mouse xenografts. Further, elevated expression of FcRn, but not of a mutated variant (H166A) that does not interact with albumin whilst retaining IgG binding [14], leads to substantially slower tumor growth rates. Collectively, our observations define a novel function for this receptor, namely as a tumor suppressor through its ability to recycle albumin.

The abnormally low levels of albumin observed in late stage cancer patients have been associated with malnutrition and inflammation, which suppress albumin synthesis by hepatocytes [23]. Inflammatory cytokines such as TNF-α contribute to decreased albumin production, and albumin levels can be used as a prognostic indicator for cancer [5]. Our observations indicating a dependency of tumor growth in mouse xenografts on albumin accumulation, combined with the correlation between hypoalbuminemia and tumor size, are consistent with earlier suggestions that tumors scavenge this protein [24]. The consumption of this protein is therefore a likely contributor to hypoalbuminemia in cancer patients. Clinically, albumin dependence of tumors has implications for the concept that nutritional supplementation may be indicated in hypoalbuminemic patients [5]. Although such intervention could relieve the symptoms associated with low circulating albumin concentrations, it may also support tumor growth by contributing to the nutrient pool.

To date, the primary focus of analyses of FcRn expression in tumors has been directed towards investigating the role that infiltrating, FcRn-positive hematopoietic cells play in increasing cross-presentation of tumor antigens in the form of immune complexes to CD8+ T cells [25]. Further, the correlation of FcRn expression levels at the tumor site with favorable prognosis has been proposed to relate to the function of this receptor in hematopoietic cells [13]. However, in addition to the immunological activity of FcRn involving IgG interactions and antigen presentation [25–27], our studies demonstrate that the levels of this receptor within the tumor cells themselves modulate cancerous growth by regulating albumin accumulation. Consequently, the use of FcRn expression levels at the tumor site as a prognostic indicator necessitates consideration of both the tumor cells and hematopoietic infiltrates.

An analysis of FcRn α-chain mRNA levels in 54 breast and 8 prostate tumor cell lines reveals that the majority (51/62; 82%) of these cells express levels of FcRn α-chain mRNA that is similar or lower than that in DU145 cells [15]. Thus, our observations in the current study raise the question as to how FcRn-expressing tumor cells such as HCC1419 overcome the limited nutrient availability that is characteristic of the tumor microenvironment to meet their metabolic needs. Interestingly, the eight FcRn-expressing cells amongst the 62 breast and prostate cell lines analyzed are not Ras-transformed [15], suggesting that other compensatory pathways exist for such cells.

It is well known that the megalin-cubilin complex, which internalizes albumin into the endocytic pathway, is expressed primarily on epithelial cells [28, 29]. This leads to the possibility that this complex may be involved in receptor-mediated uptake of albumin into tumor cells. However, analyses of the EMBL-EBI Expression database [30] indicate that HCC1419 and DU145 cells express levels of megalin and, for HCC1419 cells, of cubilin that are below the detection level, indicating that the observed effects of FcRn expression can occur independently of megalin/cubilin-mediated uptake. This is also consistent with the observations of others indicating that macropinocytosis is an important pathway for nutrient (protein) uptake into tumor cells [2].

In summary, we have demonstrated that loss of FcRn expression promotes tumor cell growth and proliferation. Our data support a model in which FcRn-mediated recycling of albumin reduces amino acid availability to fuel metabolic pathways. To date, multiple roles for FcRn have been defined in both the maintenance and induction of immunity [9, 26]. Here we reveal that this multitasking receptor acts as a tumor suppressor through its ability to regulate intracellular amino acid levels.

## MATERIALS AND METHODS

### Cell lines

The prostate cancer cell lines LNCaP, 22Rv1 and DU145 were obtained from the American Type Culture Collection (ATCC, Cat. # CRL-1740, CRL-2505 and HTB-81, respectively). The prostate cancer cell lines C4-2B and LAPC-4 were generous gifts from Dr. Nima Sharifi, Department of Cancer Biology, Cleveland Clinic, Cleveland, OH. The breast cancer cell lines SK-BR-3, BT-474 and ZR-75-1 were obtained from the ATCC (ATCC Cat. # HTB-30, HTB-20 and CRL-1500, respectively). The breast cancer cell lines HCC1419 and HCC1954 were generously provided by Drs. Adi Gazdar, John Minna and Kenneth Huffman (University of Texas Southwestern Medical Center, Dallas, TX). The human microvasculature-derived cell line, HULEC-5A, was a generous gift from F. Candal (Centers for Disease Control and Prevention). All cell lines are authenticated by short tandem repeat analyses (University of Arizona Genetics Core) on an annual basis.

LNCaP, 22Rv1, C4-2B, ZR-75-1, HCC1419 and HCC1954 cells were cultured in RPMI 1640 medium (Lonza, Walkersville, MD) containing 10% fetal bovine serum (FBS), Glutamax and 1% penicillin and streptomycin, except that 5% FBS was used for HCC1954 cells. Other cell lines were cultured as follows: DU145, Eagle’s Minimum Essential Medium (EMEM, Sigma, St. Louis, MO) with 10% FBS, Glutamax and 1% penicillin and streptomycin; SK-BR-3 cells, McCoy’s 5A medium containing 10% FBS, Glutamax and 1% penicillin and streptomycin; BT-474 cells, Hybri-Care Medium (ATCC® 46-X™) containing 10% FBS, Glutamax and 1% penicillin and streptomycin; HULEC-5A, MCDB131 medium supplemented with 10% FBS and 10 mM glutamine.

### Antibodies, reagents and fluorescent labeling

For use in immunoblotting, antibodies specific for human FcRn α-chain (Cat. # Sc271745) and β_2_-microglobulin (β_2_m) (Cat. # Sc-13565) were purchased from Santa Cruz Biotechnology. Antibodies specific for MSA/HSA (Cat. # 4929S) and β-actin (Cat. # 4970s) were purchased from Cell Signaling. Horseradish peroxidase (HRP)-conjugated goat anti-rabbit IgG and anti-mouse IgG (H1L; Cat. # 111-035-003 and 115-035-003, respectively) were from Jackson ImmunoResearch Laboratories.

The engineered human IgG1-derived antibodies, MST-HN (enhanced affinity for FcRn [18]) and H435A (no detectable binding to FcRn [19]), were expressed and labeled with Alexa Fluor 647 as described previously [31]. A bivalent nanobody (camelid VHH) specific for human FcRn [32] was produced by generating a plasmid from the patented sequence encoding two VHH domains linked by a Gly-Ser_2_-Gly_2_-Ser-Gly_4_-Ser linker. This protein was expressed with a polyhistidine tag as a periplasmically secreted protein in *E. coli* and purified using Ni^2+^-NTA-agarose [33]. The nanobody was conjugated to Alexa Fluor 647 at ratio of 1:1.5 (protein:dye).

MSA (Sigma, Cat. # A3139) was conjugated to Alexa Fluor 647 at a ratio of 1:1.5 (protein:dye). Labeled MSA was analyzed by surface plasmon resonance (BIAcore T200) to confirm that labeling had not affected binding to human FcRn, using methods analogous to those described previously [34]. Labeled MSA was further analyzed by size exclusion chromatography (GE Healthcare, Superdex 200 5/150 GL, Cat. # 28906561) to ensure that it was not aggregated.

### Plasmid construction and lentiviral transduction

Human FcRn (FCGRT) cDNA was subcloned into pCDH1-EF1-coGFP-Puro (lentiviral expression vector; System Bioscience, Cat. # CD550A-1) using designed oligonucleotide primers. Splicing by overlap extension [35] was performed to insert the H166A mutation to ablate binding to albumin [14]. All primer and construct sequences are available on request. pLKO.1 plasmid constructs containing shRNAs targeting human FcRn (shRNA-5, GCTCTTTCTGGAAGCTTTCAA; shRNA-6, CGGCGAGGAGTTCATGAATTT) were purchased from Sigma. MISSION® pLKO.1-puro Empty Vector Control Plasmid DNA (Cat. # SHC001) and MISSION® pLKO.1-puro Non-Target shRNA Control Plasmid DNA scramble (Cat. # SHC016) were purchased from Sigma.

For use in DU145 cell transductions, lentiviral particles were generated by separately co-transfecting pCDH1-empty vector (EV), pCDH1-WT-FcRn or pCDH1-H166A-FcRn constructs with the pPACKH1 plasmid packaging mix (System Biosciences SBI; Cat. # LV500A-1) into 293TN cells using Lipofectamine-LTX (Invitrogen, Cat. # 15338-100) according to the manufacturer’s protocol. For use in HCC1419 transductions, lentiviral particles were produced by separately co-transfecting pLKO.1-shRNA-5 or -6 (targeting FcRn), PLKO.1-shRNA-scramble or pLKO.1-EV constructs with the MISSION^®^ Lentiviral Packaging Mix (Sigma, Cat. # SHP001) into 293TN cells using FuGENE® 6 (Promega, Cat. # E2693) according to the manufacturer’s protocol. Forty-eight hours post-transfection, lentiviral particles were precipitated and concentrated using PEG-it™ virus precipitation solution (System Biosciences SBI, Cat. # LV810A-1) according to the manufacturer’s protocol.

DU145 cells were transduced with pCDH1-EV, pCDH1-WT-FcRn or pCDH1-H166A-FcRn lentiparticles using Transdux Virus Transduction Reagent (System Biosciences SBI, Cat. # LV850A-1) according to the manufacturer’s protocol. To obtain stable cell lines, 1 μg/ml puromycin was added to the culture medium to select for puromycin resistant clones. Transduced DU145 cells were isolated by fluorescence activated cell sorting following incubation with 5 μg/ml Alexa 647-labeled FcRn-specific nanobody to detect FcRn expression, using FcRn-expressing HCC1419 cells as a comparator. Transduced DU145 cells were subsequently plated at limiting dilution to isolate clonal cell lines.

HCC1419 cells were transduced with pLKO.1-shRNA-5, pLKO.1-shRNA-6, PLKO.1-shRNA-scramble or pLKO.1-EV lentiparticles using Transdux Virus Transduction Reagent (System Biosciences SBI, Cat. # LV850A-1) according to the manufacturer’s protocol. To select for stable cell lines, 1 μg/ml puromycin was added to the medium.

To create a shRNA-resistant FcRn plasmid (Res-FcRn), site-specific mutagenesis was performed to mutate the region targeted by shRNA-5 using pCDH1-WT-FcRn as template. The resulting plasmid was used to generate lentiviral particles for infection of the shRNA-5/HCC1419 cell line, and shRNA resistant cell lines were produced as described above.

### Analyses of FcRn levels

Cells were seeded into 24 well plates (5 × 10^5^ cells/well) overnight. Subsequently, cells were pulsed with 10 μg/ml Alexa 647-labeled human IgG1-derived MST-HN [18], 10 μg/ml Alexa 647-labeled human IgG1-derived H435A [19] or 1 μg/ml Alexa 647-labeled nanobody [32] in EMEM or RPMI1640 medium adjusted to pH 6.0 (MST-HN and nanobody bind with increased affinity to FcRn at acidic pH [18, 32]), supplemented with 10% FBS depleted of IgG, for 40 minutes at 37°C in a 5% CO_2_ incubator. Cells were washed with cold DPBS (pH 6.0), trypsinized with 0.025% trypsin (pH 6.0) and fixed using 1.7% (w/v) paraformaldehyde (pH 6.0). Cell-associated Alexa 647 levels were determined using a BD Accuri C6 Flow Cytometer. Data were analyzed using FlowJo software (FlowJo).

### Uptake and recycling assays

To measure the levels of intracellular MSA and HSA (Sigma, Cat. # A8763), DU145 and HCC1419 lines were seeded (1 × 10^6^ cells/well) in 6 well plates overnight in either EMEM or RPMI1640 medium (pH 7.4) supplemented with 10% FBS. Cells were washed with DPBS and cultured in growth medium (pH 7.4, Seahorse XF base minimal DMEM Cat. # 102353) with 100 μM glucose and 1.5 μM MSA for 0, 1, 2 or 4 hrs or 1.5 μM HSA for 0 or 2 hrs at 37°C in a 5% CO_2_ incubator. The resulting cells were washed three times using cold DPBS (pH 7.4) and lysed with RIPA buffer. For analyses of the effects of pH on albumin accumulation, the pH of the medium and DPBS were adjusted using 20 mM MES (2-(N-morpholino) ethanesulfonic acid). Lysates were collected and used in immunoblotting experiments.

To determine the pinocytic uptake rates of DU145 and HCC1419 cell lines, 5 × 10^5^ cells were pulsed for 0, 15, 30 or 60 minutes with 1.5 μM tetramethylrhodamine-labeled 70 kDa dextran (Invitrogen, Cat. # D1818) for 1 hr at 37°C in a 5% CO_2_ incubator in EMEM (pH 7.4) or RPMI1640 medium (pH 7.4). To analyze recycling rates, cells were pulsed with 20 μg/ml Alexa 647-labeled transferrin for 5 minutes at 37 °C in a CO_2_ incubator. Cells were subsequently washed with DPBS and harvested (0 minute chase) or chased in medium containing 200 μg/ml holotransferrin at 37°C in a 5% CO_2_ incubator for 15, 30 or 60 minutes. Following the pulse or pulse/chase periods, cells were harvested by trypsinization, washed and fixed with 1.7% (w/v) paraformaldehyde.

To measure the recycling rate of MSA, DU145 cells (5 × 10^5^ cells/well) were seeded in 24 well plates overnight in EMEM medium (pH 7.4) supplemented with 10% FBS. Cells were washed with DPBS and pulsed with 1.5 μM Alexa 647-labeled MSA for 2 hrs at 37°C in a 5% CO_2_ incubator in growth medium (pH 7.4, Seahorse XF base minimal DMEM Cat. # 102353) supplemented with 100 μM glucose. Cells were washed with DPBS and harvested (0 minute chase) or chased in medium containing 15 μM unlabeled MSA at 37°C in a 5% CO_2_ incubator for 15, 30 or 60 minutes. Following the pulse or pulse/chase periods, cells were harvested by trypsinization, washed and fixed with 1.7% (w/v) paraformaldehyde.

To measure the effect of pH on fluid phase uptake, DU145 and HCC1419 cells (5 × 10^5^ cells/well) were seeded in 24 well plates overnight in EMEM or RPMI1640 medium (pH 7.4) supplemented with 10% FBS. Cells were washed with DPBS (pH 7.5, pH 7.0 or pH 6.5) and pulsed with 1.5 μM Alexa 647-labeled H435A for 2 hrs at 37°C in a 5% CO_2_ incubator in growth medium (pH 7.5, pH 7.0 or pH 6.5; Seahorse XF base minimal DMEM Cat. # 102353) supplemented with 100 μM glucose. Following the pulse periods, cells were harvested by trypsinization, washed and fixed with 1.7% (w/v) paraformaldehyde.

For both pinocytic uptake and recycling assays, tetramethylrhodamine and Alexa Fluor 647 levels were determined by flow cytometry (BD LSRFortessa and BD Accuri C6 Flow Cytometer, respectively) and data analyzed using FlowJo software.

### Immunoblotting

Cells (approximately 1.5 × 10^6^/well) were grown in 6 well plates, treated as indicated in the Figure legends and harvested. Cells were lysed using RIPA buffer, and lysates (10 μg each) run on SDS-polyacrylamide gels and transferred onto Protran NC membranes (Amersham GE Healthcare, Cat. # 10600002). Membranes were immunoblotted with primary antibodies and bound antibody detected using horseradish peroxidase-conjugated secondary antibody. Bound secondary antibody was visualized using a chemiluminescent detection reagent (WesternSure PREMIUM chemiluminescent substrate, LI-COR, Cat. # 926-95000). Immunoblot images were scanned using a C-DiGit Blot Scanner and ImageJ software was used to quantitate the signals.

### Cell proliferation assay

DU145 or HCC1419 cell lines were seeded into 96 well plates (1.5 × 10^5^ cells/well) overnight. Subsequently, cells were washed with DPBS and cultured in medium (pH 7.4, Seahorse XF base minimal DMEM Cat. # 102353) supplemented with 100 μM glucose and 1.5 μM MSA (Sigma, Cat. #A3139) or 2 mM glutamine at 37°C in a 5% CO_2_ incubator for 16 hours. Cell proliferation was analyzed using the BrdU Cell Proliferation Assay Kit (Cat. # K306-200, Biovision) according to the manufacturer’s instructions. Cells were pulsed with BrdU for 4 hrs at 37°C in a 5% CO_2_ incubator. BrdU incorporation was quantitated by determining the O.D. at 450 nm.

### Intracellular glutamate assay

DU145 or HCC1419 cell lines were seeded into 6 well plates (1.6 × 10^6^ cells/well). Sixteen hours later, cells were washed three times with cold DPBS and cultured in base medium (pH 7.4, Seahorse XF base minimal DMEM Cat. # 102353) containing 100 μM glucose and 1.5 μM (100 μg/ml) MSA or 2 mM glutamine for 24 hrs at 37°C in a 5% CO_2_ incubator. Cells were collected and lysed with 1% Triton X-100. Cell lysates were deproteinized using the Deproteinizing Sample Preparation Kit (Cat. # 808-200, BioVision) and intracellular glutamate levels measured using the BioAssay Systems EnzyChrom™ Glutamate Assay Kit (BioAssay Systems, Cat. # EGLT-100) according to the manufacturer’s instructions.

### Mouse xenograft studies

BALB/c scid mice were purchased from Jackson Laboratories, bred and maintained in specific pathogen-free housing. All mouse procedures were carried out in accordance with protocols approved by the IACUC at Texas A&M University. HCC1419 cells (7×10^6^/mouse) and DU145 cells (5×10^6^/mouse) were implanted subcutaneously in the flanks into 6-8 week old female and male mice, respectively. Cells were suspended in a 1:1 mixture of medium:Matrigel (Corning, Cat. # 354234). For estrogen receptor-positive HCC1419 cells [36], mice were implanted subcutaneously in the neck with 0.72 mg 60 day release estradiol pellets (Innovative Research of America; Cat. # SE-121) three days prior to cell implantation. Tumor size was determined using calipers every three days. Mice were bled retroorbitally at the indicated times. At the end of each experiment, mice were perfused with PBS containing 10 U/ml heparin and tumors isolated for immunohistochemistry or immunoblotting.

For use in immunoblotting, tumors extracted from mice were lysed in RIPA buffer using a kit from the Peglab (precellys ceramic kit 2.8 mm, Cat. # 91-PCS-CK28) and minilys homogenizer (Bertin Technologies). Resulting lysates were subjected to centrifugation. Supernatants were collected and protein concentrations were determined using the BCA protein assay kit (Pierce, Cat. # 23225).

### Determination of serum albumin and alanine aminotransferase levels

Albumin concentrations in mouse serum samples were determined using ELISAs with goat anti-mouse albumin (Abcam, Cat. # ab19194) as coating antibody and HRP-conjugated goat anti-mouse albumin antibody (Abcam, Cat. # ab19195) as detection antibody. Mouse serum albumin (Sigma, Cat. # A3139) was used to generate a standard curve. Alanine aminotransferase levels in serum samples were quantitated using a commercially available kit (Sigma, Cat. # MAK052).

### Immunohistochemistry

HCC1419 tumors were snap-frozen in O.C.T. Compound (Fisher Scientific, Cat. # 4584). Samples were stored at -80°C prior to preparation of 10 μm thick cryosections using an HM 550 P cryostat microtome (Microm GmbH, Cat. # 956424). Sections were fixed with 4% paraformaldehyde (Electron Microscopy Sciences, Cat. # 19200), permeabilized with 0.05% Triton X-100 (Fisher Scientific, Cat. # BP151-100), blocked with IgG from goat serum (Sigma, Cat. # I5256), followed by blocking of endogenous biotin or biotin-binding activity (Life Technologies, Cat. # 004303). Sections were then incubated with FcRn-specific antibody (ADM31, Aldevron Freiburg GmbH, Cat. # AK2154/01.1) overnight at 4°C and the same concentration of mouse IgG2b (Invitrogen, Cat. # 02-6300) was used for isotype control slides.

Following overnight incubation, sections were incubated with biotin-SP-conjugated AffiniPure goat anti-mouse IgG (Jackson ImmunoResearch Laboratories, Cat. # 115-065-146) and bound conjugate detected using Alexa Fluor 647-conjugated neutravidin (Molecular Probes, Cat. # A2666). Neutravidin was conjugated with Alexa Fluor 647 succinimidyl ester (Molecular Probes, Cat. # A20006).

Stained sections were mounted using hard set mounting medium with DAPI (Vector Laboratories, Cat. # H-1500) and imaged using a Zeiss Axiovert 200M inverted fluorescent microscope with a Zeiss 20x/0.8NA Plan Apochromat objective (Carl Zeiss Microscopy, Cat. # 420651-9911). Images were acquired with filter sets for DAPI and Alexa Fluor 647 from Semrock (Cat. # DAPI-5060C-ZHE and Cy5-4040C-ZHE, respectively), using a Hamamatsu Orca ER CCD camera (Hamamatsu Photonics Systems, Model C4742-95-12ER).

Images were acquired using MIAcquire, a custom software developed and written in our laboratory in C programming language. This software directly commands all hardware components for the acquisition. Acquired images were visualized in real-time via a connection to the analysis software MIATool [37]. MIATool is written in Java and was used for analysis and image processing for all of the acquired images. Intensities of the images were linearly adjusted to the same level. For the overlay images, DAPI and FcRn were pseudocolored red and green, respectively.

For quantitation of signals obtained during immunohistochemical analyses, 5-9 images per section were acquired and median fluorescence intensities determined. Median intensity values were compared with the signal obtained from HCC1419 tumor sections, using data obtained from two independent experiments.

### Statistical analyses

Statistically significant differences were determined using one-way ANOVA (*in vitro* assays) or two-way ANOVA (tumor xenografts) followed by Tukey post-hoc test, and *p* values of less than 0.05 were considered to be significant. The correlation coefficients for albumin levels vs. tumor size were compared using z scores after applying a Fisher r-to-z transformation.

## SUPPLEMENTARY MATERIALS FIGURES


