# Supplementary material for: Loss of expression of the recycling receptor, FcRn, promotes tumor cell growth by increasing albumin consumption
Source: Oncotarget. 2016 Dec 10;8(2):3528–41. doi: 10.18632/oncotarget.13869 (PMC5356901; doi:10.18632/oncotarget.13869)
Supplement: Supplementary file 1 [file oncotarget-08-3528-s001.pdf]

Loss of expression of the recycling receptor, FcRn, promotes tumor cell growth by increasing albumin consumption

SUPPLEMENTARY FIGURES

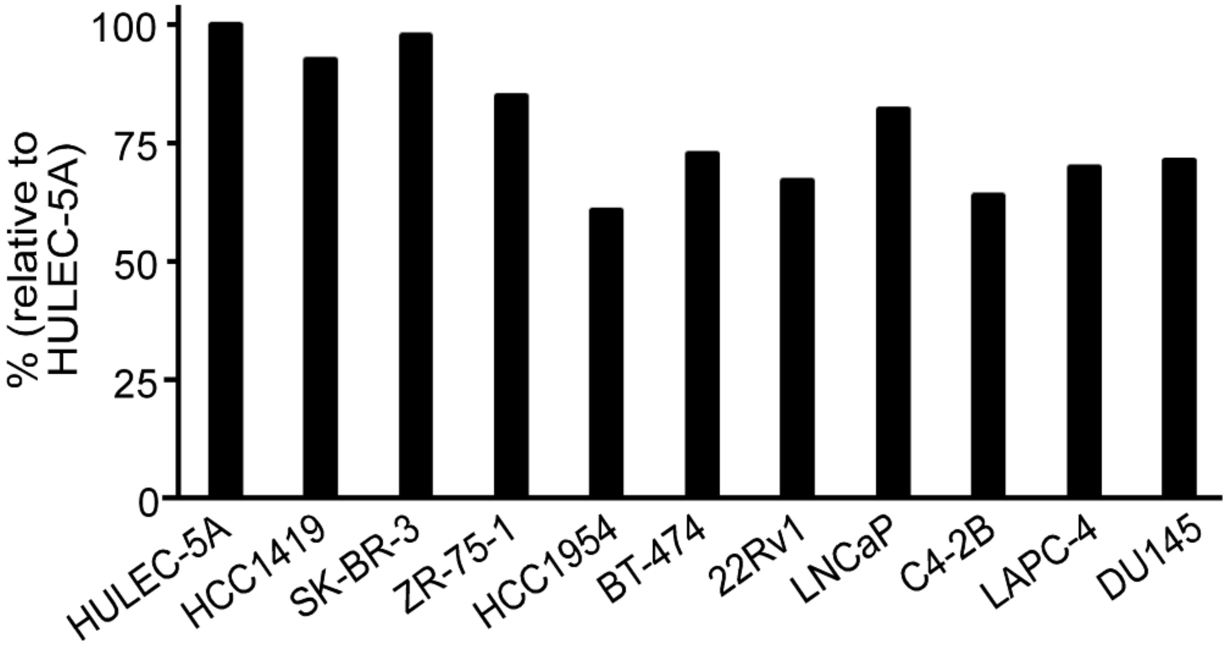

Supplementary Figure S1: Quantitation of β_2m expression from immunoblotting of tumor cell lysates. β_2m expression levels in the endothelial cell line, HULEC-5A, are taken as 100%. Data are representative of two independent experiments.

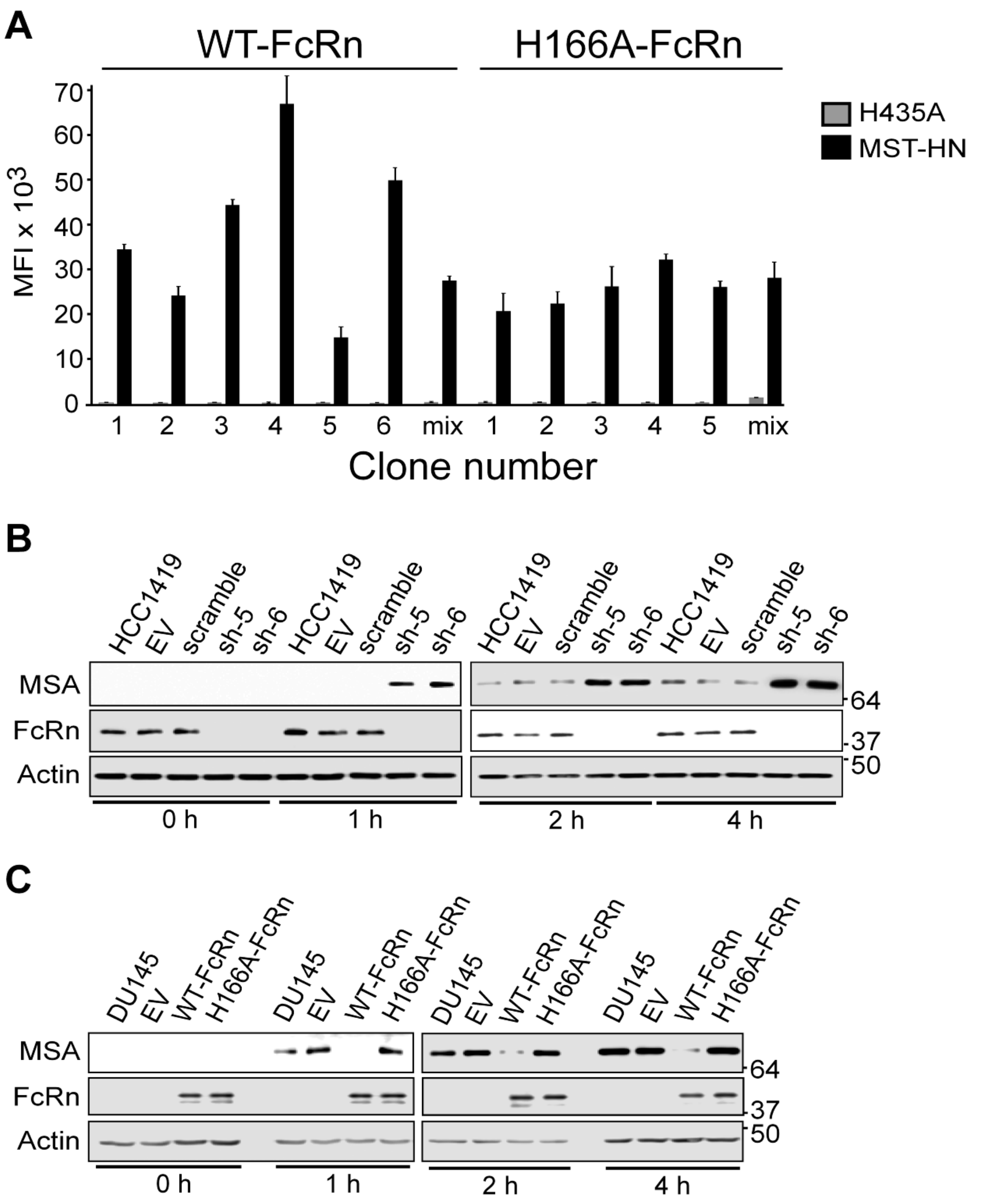

Supplementary Figure S2: Generation and characterization of clonal DU145 lines and time course of MSA uptake by HCC1419 and DU145 cell lines. A. Clonal DU145 cell lines expressing WT-FcRn or H166A-FcRn were pulsed with 10 µg/ml Alexa 647-labeled MST-HN or H435A for 40 minutes at 37°C in medium (pH 6.0). Data for the corresponding polyclonal cell lines are also shown (mix). Alexa 647 levels (MFI) were determined using flow cytometry and mean values for triplicate samples are shown. Error bars represent S.D. **B, C.** HCC1419 (B) and DU145 (C) cell lines were pulsed with 1.5 µM mouse serum albumin (MSA) for 0, 1, 2 or 4 hrs at 37°C at pH 7.4. Immunoblotting of cell lysates using antibodies specific for MSA, FcRn α-chain and β-actin was performed. Cropped images are shown with molecular weights (kDa) on the right. Data are representative of two independent experiments.

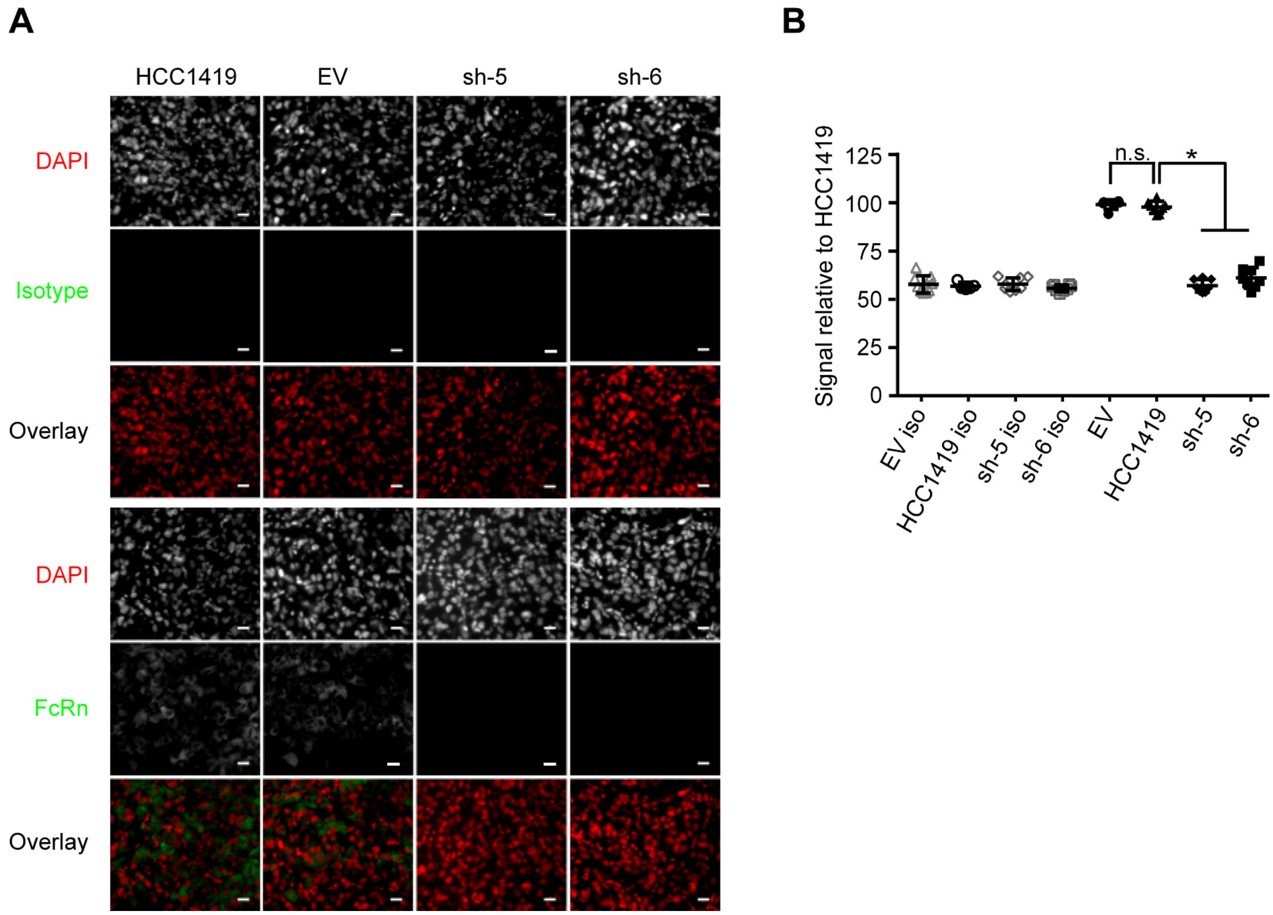

Supplementary Figure S3: Immunohistochemical analyses of FcRn expression in tumors extracted from mice. A. Tumors were extracted from mice at day 31 to detect FcRn expression (fluorescence for FcRn and DAPI are pseudocolored green and red, respectively). Representative images for tumors from mice within each group are shown. Bars = 20 µm. **B.** Quantitation of FcRn-specific staining of tumor sections relative to staining levels for HCC1419 (parent cell line) tumors. Iso denotes isotype control. Error bars represent S.D. Significant differences are indicated by * (one-way ANOVA, $p < 0.05$). n.s. = no significant difference.

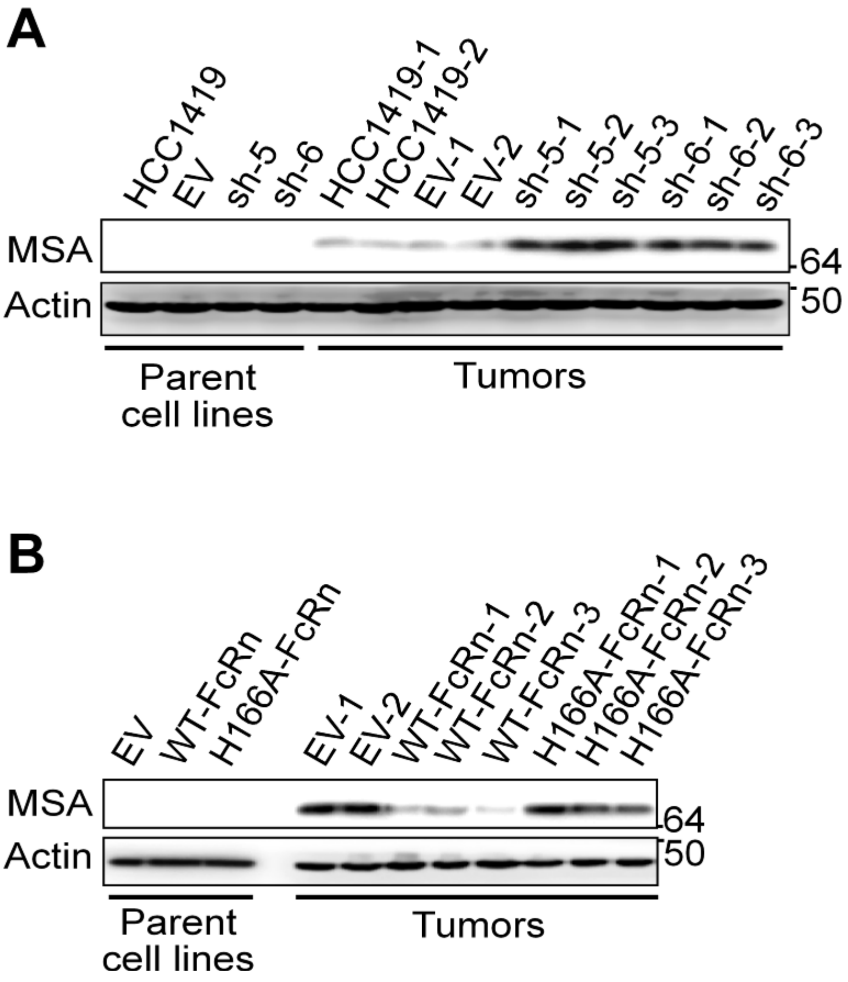

Supplementary Figure S4: The expression level of FcRn controls the intracellular levels of albumin in tumor xenografts. A, B. Immunoblotting of lysates of tumors extracted from mice at the end of the xenograft experiments for HCC1419 cell lines (A) and DU145 cell lines (B), using antibodies specific for human FcRn α-chain and β-actin. Cropped images are shown with molecular weights (kDa) on the right. Data are representative of two independent experiments.

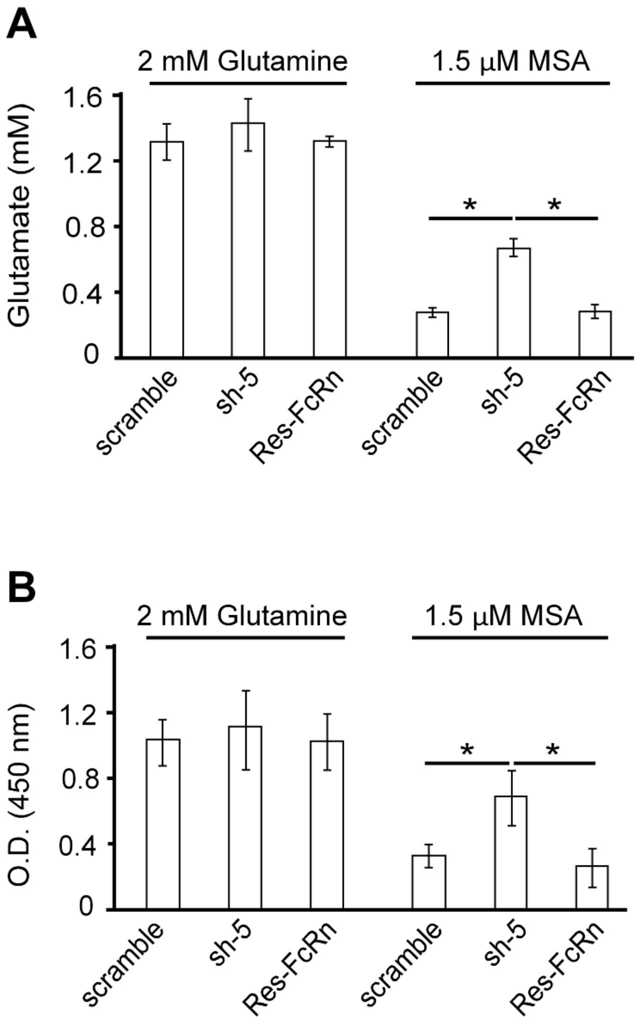

Supplementary Figure S5: Functional FcRn can be restored in the HCC1419/shRNA-5 (sh-5) cell line by transduction with a shRNA resistant, FcRn construct (Res-FcRn). A. HCC1419 cell lines lines transduced with scrambled shRNA (scramble), shRNA-5 (sh-5) or sh-5 plus shRNA resistant FcRn (Res-FcRn) were cultured in base medium containing 100 μM glucose and 1.5 μM MSA or 2 mM glutamine for 24 hrs. Cells were lysed and glutamate levels determined. Mean values (mM glutamate) of triplicate samples are shown. **B.** HCC1419 cell lines were cultured in base medium containing 100 μM glucose and 1.5 μM MSA or 2 mM glutamine for 16 hours. Cells were pulsed with BrdU for 4 hrs and BrdU levels determined. Mean O.D. (450 nm) values for triplicate samples are shown. Error bars represent S.D. Significant differences are indicated by * (one-way ANOVA, $p < 0.05$). Data are representative of two independent experiments.
